# Stereo-Specific Modulation of the Extracellular Calcium-Sensing Receptor in Colon Cancer Cells

**DOI:** 10.3390/ijms221810124

**Published:** 2021-09-19

**Authors:** Martin Schepelmann, Nadja Kupper, Marta Sladczyk, Bethan Mansfield, Teresa Manhardt, Karina Piatek, Luca Iamartino, Daniela Riccardi, Benson M. Kariuki, Marcella Bassetto, Enikö Kallay

**Affiliations:** 1 Center for Pathophysiology, Infectiology and Immunology, Institute for Pathophysiology and Allergy Research, Medical University of Vienna, Waehringer Guertel 18-20, 1090 Vienna, Austria; nadja.kupper@meduniwien.ac.at (N.K.); marta.sladczyk@gmail.com (M.S.); teresa.manhardt@meduniwien.ac.at (T.M.); karina.piatek@meduniwien.ac.at (K.P.); luca.iamartino@unifi.it (L.I.); 2 Cardiff School of Biosciences, Cardiff University, Museum Avenue, Cardiff CF10 3AX, UK; MansfieldB@cardiff.ac.uk (B.M.); Riccardi@cardiff.ac.uk (D.R.); 3 Department of Experimental and Clinical Biomedical Sciences, University of Florence, Viale Pieraccini 18, 50139 Florence, Italy; 4 School of Chemistry, Cardiff University, Main Building, Park Place, Cardiff CF10 3AT, UK; KariukiB@cardiff.ac.uk; 5 Department of Chemistry, Faculty of Science and Engineering, Swansea University, Singleton Park Campus, Swansea SA2 8PP, UK; marcella.bassetto@swansea.ac.uk

**Keywords:** calcium-sensing receptor, enantiomer, calcimimetic, calcilytic, colon cancer, stereospecificity, HT-29, IL-8, inflammation

## Abstract

Pharmacological allosteric agonists (calcimimetics) of the extracellular calcium-sensing receptor (CaSR) have substantial gastro-intestinal side effects and induce the expression of inflammatory markers in colon cancer cells. Here, we compared the effects of both CaSR-specific (*R* enantiomers) and -unspecific (*S* enantiomers) enantiomers of a calcimimetic (NPS 568) and a calcilytic (allosteric CaSR antagonists; NPS 2143) to prove that these effects are indeed mediated via the CaSR, rather than via off-target effects, e.g., on β-adrenoceptors or calcium channels, of these drugs. The unspecific *S* enantiomer of NPS 2143 and NPS *S-*2143 was prepared using synthetic chemistry and characterized using crystallography. NPS *S*-2143 was then tested in HEK-293 cells stably transfected with the human CaSR (HEK-CaSR), where it did not inhibit CaSR-mediated intracellular Ca^2+^ signals, as expected. HT29 colon cancer cells transfected with the CaSR were treated with both enantiomers of NPS 568 and NPS 2143 alone or in combination, and the expression of CaSR and the pro-inflammatory cytokine interleukin 8 (IL-8) was measured by RT-qPCR and ELISA. Only the CaSR-selective enantiomers of the calcimimetic NPS 568 and NPS 2143 were able to modulate CaSR and IL-8 expression. We proved that pro-inflammatory effects in colon cancer cells are indeed mediated through CaSR activation. The non-CaSR selective enantiomer NPS *S-*2143 will be a valuable tool for investigations in CaSR-mediated processes.

## 1. Introduction

The extracellular calcium-sensing receptor (CaSR) is a class C G protein-coupled receptor (GPCR) [1]. Its main and best-described physiological role is monitoring and regulating free ionized blood calcium (Ca^2+^) concentration [2]. However, the CaSR has several other functions independent of calcium homeostasis. It regulates physiological and pathophysiological processes, such as inflammation, airway constriction, renal and intestinal water transport, cardiovascular effects, neuronal development and function, and entero-endocrine hormone secretion, etc., in a tissue- and ligand-dependent manner [3]. The CaSR is a multimodal sensor, which has multiple ligand-binding sites for the different orthosteric and allosteric ligands, including divalent and trivalent cations, protons, L-amino acids, polyamines, aminoglycosides, or polycationic molecules [4]. These ligands can activate different G proteins (G_q/11_, G_i/o_, G_12/13_, and in some cases even G_s_). Besides the natural ligands, several pharmacological modulators of the CaSR have been developed, drugs that either mimic (calcimimetics) or antagonize (calcilytics) the effects of extracellular Ca^2+^ on the CaSR, as reviewed in [5].

As most of the ligands of the CaSR are able to act through other molecules (receptors and channels), it is very difficult to prove that a certain signaling pathway is indeed mediated by the CaSR. For instance, polycationic peptides can insert into membranes and thus alter G protein activity; aminoglycosides can block voltage-sensitive Ca^2+^ channels; Ca^2+^ itself can also signal through various channels; calcimimetics were originally derived from Ca^2+^ channel blockers, while the amino-alcohol class of calcilytics is similar to beta-blockers. Thus, it is difficult to determine whether a certain effect of a CaSR ligand is indeed mediated via the CaSR or via other routes.

The role of the CaSR in the intestine is not yet clear. Its effect on regulating intestinal inflammatory processes is a point of contention [6]. Several authors suggested a role in preventing inflammation [7,8], while we found a rather pro-inflammatory effect [9,10]. In addition, clinically used calcimimetics have severe gastrointestinal side effects [11], suggesting a role for the intestinal CaSR in mediating these adverse responses. Therefore, in the present study, we tested whether the CaSR has pro- or anti-inflammatory effects, by using positive (NPS 568) and negative (NPS 2143) allosteric modulators of the CaSR and comparing their effects on the expression of the inflammation marker interleukin 8 (IL-8), as several studies suggested that activation of the CaSR leads to the inhibition of IL-8 secretion in colon cancer cells (as reviewed in [6]). To ensure that the results are indeed specific for the CaSR, we used the *R* and *S* enantiomers of both modulators. Enantiomers are “mirror images” of a chemical structure, which only differ in the configuration (stereochemical orientation) of a chiral, i.e., asymmetric atom. The chemical structures of both NPS 568 and NPS 2143 contain one such asymmetric carbon. In both cases, the respective *R* enantiomer binds with much higher affinity to the CaSR than the *S* enantiomer. Previous studies have shown that the *R* enantiomer of NPS 568 is 10-fold more potent than the corresponding *S* enantiomer in inhibiting parathyroid hormone (PTH) secretion from bovine parathyroid cells [12]. To investigate whether the pro-inflammatory effects of calcimimetics in the colon are indeed mediated via the CaSR, we used the colon cancer cell line HT29 transfected either with the CaSR (HT-29^CaSR-GFP^) or with the empty vector (HT29^GFP^). These transfections are necessary because colonic epithelial cells, during transformation into cancer cells, lose their native CaSR expression. As there are no reliable native colon epithelial cell lines available, we used these modified cancer cells to study the effect of the CaSR in colonic epithelial cells in vitro where we had already determined that CaSR expression and stimulation using *R-*568 leads to massive changes in their gene expression pattern, including a strong upregulation of pro-inflammatory genes [9].

We were able to prove unequivocally that, in this cell model, the activation of the CaSR by Ca^2+^ or NPS *R-*568 induced the expression of the inflammation marker IL-8, while the calcilytic NPS *R*-2143 was able to prevent this induction. To the best of our knowledge, this is the first study to use both the *R* and *S* forms of a calcimimetic and calcilytic to identify whether a pharmacological effect is indeed mediated via the CaSR. As the non-CaSR selective enantiomer of NPS 2143 (NPS *S-*2143) is not available commercially, we also describe the synthetic route and characterization of this valuable pharmacological tool.

## 2. Results

As NPS *S-*2143 could not be obtained from any other source, we synthetized and evaluated it in-house.

### 2.1. Synthetic Chemistry

**Figure 1:**
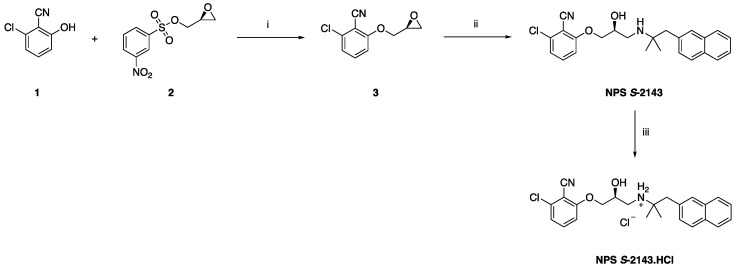
Preparation of **NPS *S*-2143** and its hydrochloride salt. Reagents and conditions: (**i**) K_2_CO_3_, acetone, reflux, o.n. (98%); (**ii**) [1,1-dimethyl-2-(2-naphthalenyl)ethyl]amine, EtOH, 80 °C, 72 h (73%); (**iii**) conc. HCl, MeOH, r.t., 1 h (quantitative).

The pure (*S*) enantiomer of NPS 2143 (NPS *S*-2143) was prepared following a previously described approach for the synthesis of the (*R*) enantiomer [13], according to the synthetic route summarized in Figure 1.

Briefly, substituted phenol **1** was deprotonated in refluxing acetone, using an excess of potassium carbonate. Commercially available (*S*)-nosyl epoxide **2** was then added at r.t., and the mixture was refluxed in acetone overnight, to afford the pure intermediate **3** in high yield after flash column chromatography purification. The epoxide ring in **3** was then regioselectively opened with [1,1-dimethyl-2-(2-naphthalenyl)ethyl]amine at the less hindered position, by heating the two reagents in anhydrous EtOH at 80 °C in a sealed tube for 72 h, to afford the enantiomerically pure product NPS *S*-2143 in good yield after flash column chromatography purification. The pure hydrochloride salt of NPS *S*-2143 was finally obtained by treating NPS *S*-2143 with an excess of a concentrated hydrochloric acid solution, while stirring in MeOH at room temperature for 1 h.

### 2.2. Crystal Structure of NSP S-2143.HCl

**Figure 2:**
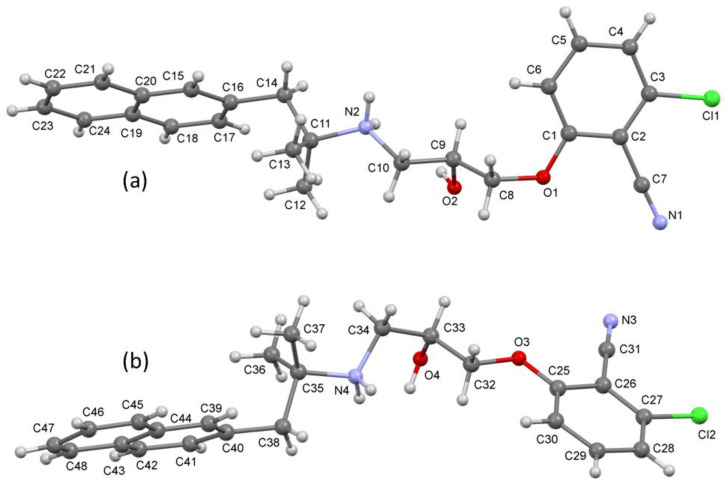
Ball and stick 3D representation of the molecular structure of NPS *S*-2143, confirming the absolute (*S*) configuration at the asymmetric carbon, showing a comparison of the two independent cations of NPS *S*-2143 (**a**,**b**) comprising the crystal, viewed roughly along the C-OH bond, e.g., showing the variation in the torsion angles between the corresponding N2-C10-C9-C8 (158.5(4)°) and N4-C34-C33-C32 (−65.4(5)°) bonds. Colors denote different atoms: grey = carbon, red = oxygen, green = chlorine, blue = nitrogen, light grey = hydrogen.

The crystal structure of NPS *S*-2143.HCl was determined by small molecule crystallography, which confirmed the desired (*S*) absolute configuration of the chiral center. In addition to two independent chloride anions, the asymmetric unit of the crystal structure comprises two independent cations, C1-C24, N1, N2, O1, O2, Cl1 (Figure 2a) and C25-C48, N3, N4, O3, O4, Cl2 (Figure 2b). Both are the cations of NPS *S*-2143 (with chiral centres located on atoms C9 and C33), which differ in their assumption of different conformations, as illustrated by Figure 2 and the torsion angles in Table S1. When packed into the crystal, aromatic rings of the chlorobenzonitrile moieties of neighbouring cations are stacked in the a-axis direction with centroid-to-centroid separation of ca. 3.6 Å. The molecules involved are also bridged by N-H…Cl and O-H…Cl hydrogen bonds with each chloride ion interacting with two N-H and one O-H groups (Figure S1 and Table S2).

### 2.3. Enantiospecific Inhition of the CaSR via NPS 2143

**Figure 3:**
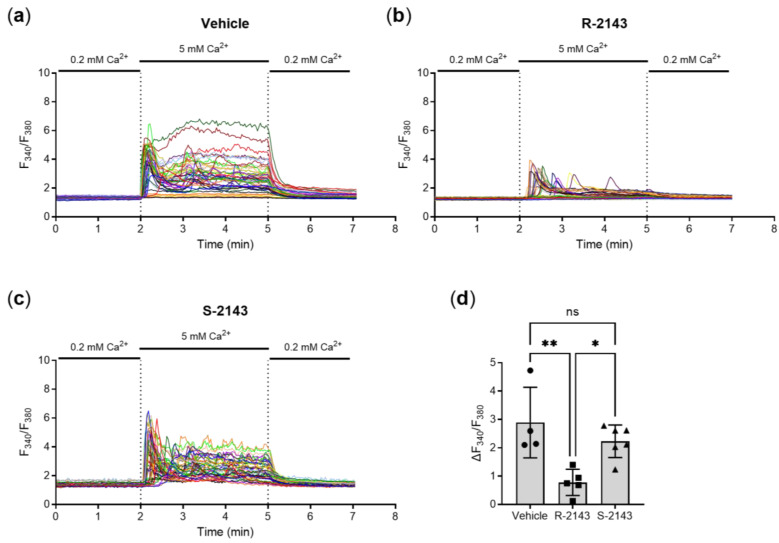
Example traces of the intracellular Ca^2+^ response (shown as fluorescence ratio F340/F380 of the Ca^2+^-dye fura-2 AM) of HEK-293 cells stably transfected with the human calcium-sensing receptor CaSR in response to 5 mmol/L Ca^2+^, 10 min pre-incubated and perfused with either (**a**) vehicle control (0.1% DMSO), (**b**) 100 nmol/L *R-*2143, or (**c**) 100 nmol/L *S-*2143; each colored line represents a single cell. (**d**) Average maximum intracellular Ca^2+^ response per cell subtracted from its baseline response for all experiments (*N* = 4–6, each *N* represents the average response of > 50 cells). Mean ± SD, one-way ANOVA with Tukey post-test, ** *p* < 0.01, * *p* < 0.05.

With NPS *S-*2143 now available, we proceeded to test the synthetized compound in vitro by individual-cell Ca^2+^-imaging performed on HEK-293 cells stably transfected with the human CaSR, which is the most common cell model for evaluating the receptor and agents targeting it. Pre-incubation of the cells with a concentration of 100 nmol/L of NPS *R-*2143 (the selective enantiomer of the compound) significantly suppressed the intracellular Ca^2+^-response elicited by 5 mmol/L extracellular Ca^2+^, while the newly synthetized (unselective) NPS *S-*2143 had no such effect (Figure 3a–d). Together, these results showed that the clean *S* enantiomer of NPS 2143 is indeed not active on the CaSR. It could thus be used for the following specificity experiments in colorectal cancer cell.

### 2.4. CaSR Gene Induction and Pro-Inflammatory Responses in Colon Cancer Cells Are Mediated through the CaSR

Having established the different activities of the two enantiomers of NPS 2143, we evaluated the specificity of the enantiomers of calcimimetics and calcilytic and their combination in a cell model of colorectal cancer. Here, we used HT29 colon cancer cells, which were modified to stably express the CaSR via lentiviral infection (HT29^CaSR-GFP^) using empty vector infected cells as the control (HT29^GFP^).

To test directly whether the calcimimetic-induced effects on *CaSR* expression were indeed mediated via specific activity of the drug on the receptor or whether unspecific effects of the drug were (partly) responsible as well, we performed a series of tightly controlled single and combination treatment experiments with the selective and non-selective enantiomers of the calcimimetic NPS 568 and the calcilytic NPS 2143. To ensure proper interaction of the calcilytic with the receptor, cells were always pre-treated with NPS 2143 (or vehicle) for 30 min before their treatment with NPS 568 (if applicable). The total treatment time after the addition of NPS 568 was 4 h. Here, we used 1 µmol/L of each compound, as the initial results using NPS *R-*568 and NPS *R-*2143 in these transfected colon cancer cells were obtained using drug concentrations that were previously deemed to be CaSR-selective [5,9,14,15,16].

The CaSR has a rather unique property in that its expression increases in response to stimulation (rather than decreases as most receptors do due to desensitization and internalization). This process can be observed directly at the cell membrane, where it is called agonist-driven insertional signaling (ADIS) [17], but also at the mRNA level.

**Figure 4:**
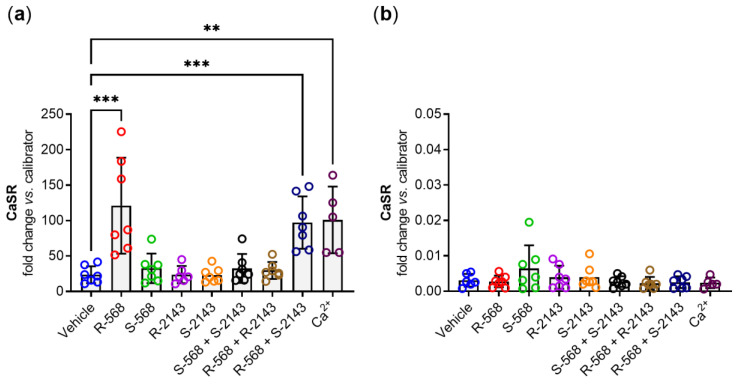
*CaSR* gene expression of (**a**) HT29^CaSR-GFP^ and (**b**) HT29^GFP^ cells (note the 5000× smaller scale of the *y*-axis compared to (**a**) treated for 4 h with either vehicle control (0.1% DMSO) or single treatments with 1 µmol/L of NPS *R*-568, NPS *S-*568, NPS *R*-2143, or NPS *S-*2143; double treatments with 1 µmol/L of each NPS *S-*568 + S2143, NPS *R*-568 + *S-*2143, or NPS *R*-568 + *R*-2143; or with 5 mmol/L of the orthosteric CaSR agonist Ca^2+^). *N* = 5–7, Mean ± SD, one-way ANOVA with Dunnett post-test vs. vehicle, *** *p* < 0.001, ** *p* < 0.01.

Treatment of HT29^CaSR-GFP^ cells with *R-*568 evoked a large (~5-fold) increase in *CaSR* gene expression after 4 h of treatment. In contrast, the non-selective enantiomer of the calcimimetic, *S-*568, did not have any effect on *CaSR* gene expression. Neither the selective nor the non-selective enantiomers of NPS 2143 had any effect on *CaSR* gene expression, either by themselves or in combination. Importantly, pre-treatment with the selective NPS *R-*2143 completely abolished the *CaSR* gene induction by NPS *R-*568. On the other hand, pre-treatment with the non-CaSR-selective enantiomer NPS *S-*2143 did not have any effect on NPS *R-*568-induced *CaSR* gene expression. The most prominent orthosteric agonist of the CaSR, Ca^2+^, also upregulated *CaSR* gene expression, and was used as a positive control (Figure 4a). None of the compounds or their combinations had any effect on *CaSR* expression levels in HT29^GFP^ cells, which was expected as the expression levels of the *CaSR* in the non-CaSR transfected HT29^GFP^ cells are negligible (Figure 4b). Taken together, these results showed that both NPS 568 and NPS 2143 affected *CaSR* gene expression in a highly enantiospecific manner. This strongly indicates that the induction of *CaSR* gene expression is indeed induced directly via the CaSR itself.

Next, we wanted to assess whether the observed enantiospecific effects of CaSR-modulation could be observed on a CaSR-influenced effector gene. As mentioned before, we have recently shown that the modulation of the CaSR with NPS *R*-568 led to dramatic changes in their gene expression patterns. One of the most strikingly upregulated families of genes were involved in inflammation. Thus, we investigated the effect of the above-mentioned single enantiomers and their combinations on *IL-8* gene expression, as one of the most prominent members of the previously observed pro-inflammatory genes.

**Figure 5:**
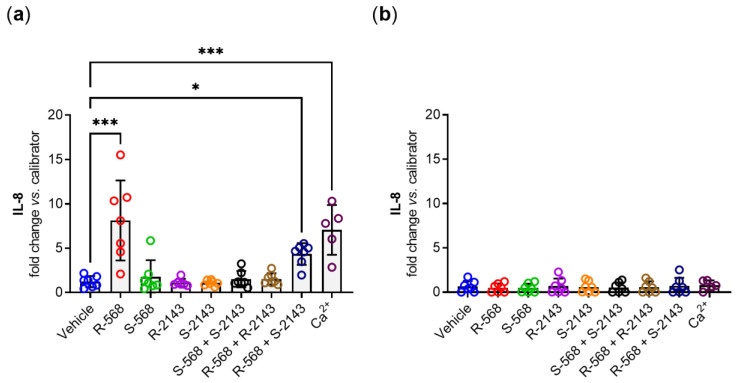
*Interleukin 8* (*IL-8*) gene expression in (**a**) HT29^CaSR-GFP^ and (**b**) HT29^GFP^ cells treated for 4 h with either vehicle control (0.1% DMSO) or single treatments with 1 µmol/L of *R-*568, *S-*568, *R-*2143, or *S-*2143; double treatments with 1 µmol/L of each *S-*568 + S2143, *R-*568 + *S-*2143, or *R-*568 + *R-*2143; or with 5 mmol/L of the orthosteric CaSR agonist Ca^2+^. *N* = 5–7, Mean ± SD, one-way ANOVA with Dunnett post-test vs. vehicle, *** *p* < 0.001, * *p* < 0.05.

We observed the same pattern for *IL-8* gene expression as for *CaSR* in HT29^CaSR-GFP^ cells. NPS *R-*568 induced a strong upregulation of *IL-8*, while the unspecific NPS *S-*586 showed no effect whatsoever. Both enantiomers of the calcilytic NPS 2143 did not affect *IL-8* gene expression, and neither did the combination of the two unselective enantiomers NPS *S-*568 and NPS *S-*2143. Again, pre-treatment of the cells with NPS *R-*2143 inhibited NPS *R-*568-induced *IL-8* gene expression, while NPS *S-*2143 was not able to suppress this upregulation. The orthosteric CaSR agonist Ca^2+^ also induced a robust upregulation *of IL-8* gene expression (Figure 5a). In HT29^GFP^ cells, none of the substances led to any change in *IL-8* gene expression compared with the vehicle control (Figure 5b).

**Figure 6:**
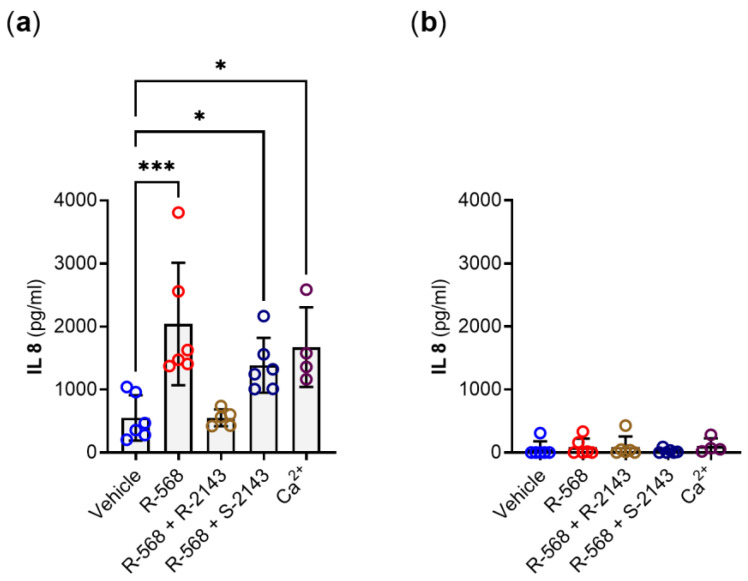
IL-8 protein (ELISA) in cell culture medium of (**a**) HT29^CaSR-GFP^ and (**b**) HT29^GFP^ cells treated for 4 h with either vehicle control (0.1% DMSO); 1 µmol/L of *R-*568; double treatments with 1 µmol/L of each *R-*568 + *S-*2143, or *R-*568 + *R-*2143; or with 5 mmol/L of the orthosteric CaSR agonist Ca^2+^. *N* = 4–6, Mean ± SD, one-way ANOVA with Holm–Sidak post-test vs. vehicle, *** *p* < 0.001, * *p* < 0.05.

Finally, we investigated whether changes in *IL-8* gene expression were mirrored by changes at the secreted protein level. We, therefore, performed an ELISA experiment on cell culture supernatants from treated HT29^CaSR-GFP^ and HT29^GFP^ cells and observed the same effect on the protein level as we did on the mRNA level. NPS *R-*568 and 5 mmol/L Ca^2+^ induced upregulation of IL-8 secretion by the HT29^CaSR-GFP^ cells. The pre-incubation with NPS *R-*2143 prevented this upregulated secretion, while the unspecific NPS *S-*2143 did not (Figure 6a). No effects on IL-8 secretion were observed in HT29^GFP^ cells lacking the CaSR (Figure 6b). Interestingly, baseline IL-8 levels were already higher in vehicle-treated HT29^CaSR-GFP^ cells than in HT29^GFP^ cells.

## 3. Discussion

We were able to prove unequivocally that the CaSR mediates the Ca^2+^- and NPS *R-*568-induced increase in the expression of the inflammation marker IL-8 in HT29^CaSR-GFP^ cells. To verify this, we used both the *R* and *S* enantiomers of the calcimimetic NPS 568 and of the calcilytic NPS 2143 and compared their effects in the isogenic cell lines HT29^CaSR-GFP^ and HT29^GFP^, differing only in the expression of CaSR. As, to our knowledge, no *S* enantiomer of NPS 2143 was available, we synthesized it and thus obtained a very useful tool for further pharmacological studies on the CaSR.

After ensuring that the synthesized NPS *S*-2143 is indeed significantly less active than *R-*2143 in preventing extracellular Ca^2+^-induced intracellular Ca^2+^ release, we assessed the effectiveness of the modulators in regulating *CaSR* expression, a well-known effect of these compounds. Indeed, extracellular Ca^2+^ and the well-characterized calcimimetic NPS *R-*568 significantly induced *CaSR* expression, which was inhibited by *R-*2143. None of the *S* enantiomers affected *CaSR* expression, proving that the cells were responsive only to the *R* modulators. In the same cells, we measured the IL-8 expression at both the mRNA and protein levels. Numerous studies have shown that IL-8 is a pro-inflammatory cytokine [18] in the intestine [19,20], and thus a valid marker for a pro-inflammatory response in our cell model.

Therefore, we needed to prove that our contradictory observation [9] that the activation of the CaSR induces IL-8 expression is real, and indeed mediated by the CaSR. The results of the Iamartino study [9] were already very convincing, as the observed upregulation of several inflammatory markers was seen only in the cells expressing the CaSR but not in the cells with undetectable CaSR levels. While the observation that *R-*568 induced the expression of these markers suggested that the CaSR mediates this effect, we needed unequivocal proof. Indeed, we could show that only the actively binding *R-*568, at the same concentration of 1 µmol/L as used in the aforementioned study, induced the expression of IL-8, and NPS *S-*568 was unable to do so. The effect of NPS *R-*568 was inhibited by NPS 2143 in a similar stereospecific manner: only *R-*2143 prevented the *R-*568-dependent induction, whereas NPS *S-*2143 did not.

Several studies suggested that calcilytics could be used in the treatment of inflammatory diseases, such as asthma [14,21]. Thus, the newly synthesized *S-*2143 could become a highly useful tool in experiments of proof of principle in other tissues and organs as well.

The first generation of calcimimetics, such as NPS *R-*568, has one chiral carbon and acts stereoselectively on the CaSR. Regardless of the parameter assessed, the *R* enantiomer was always more potent (10- to 100-fold, depending on the measured variable) compared to the *S* enantiomer. At concentrations that maximally activate the CaSR, NPS 568 inhibits ion channels, but this effect is not stereospecific, meaning that both the *R* and *S* enantiomers have similar effects [22]. The calcilytic NPS 2143 also acts on the CaSR in a stereoselective manner and again, the *R* enantiomer is more potent [12,22]. Thus, testing that the compounds used in our experiment produce the expected effect in a stereospecific manner ascertained that the effect was CaSR-mediated. Moreover, none of the compounds had any effect on *IL-8* (or *CaSR*) expression in the HT29^GFP^ cells lacking detectable levels of endogenous CaSR, suggesting that at the concentrations used, the observed effects were not due to the activation of any other possible targets (e.g., voltage-gated Ca^2+^ channels).

To understand the role of the CaSR in a cell is demanding, because Ca^2+^, its main physiological ligand, signals not only through the CaSR, but also by binding to other molecules (e.g., ion channels). Therefore, it is important to combine the right pharmacological approach with the most appropriate experimental design. However, this is challenging in cells where the primary role of the receptor is not clear and Ca^2+^ affects numerous biological processes. The biological process we had to test was given (regulation of the expression of IL-8) and the specificity of the drugs used in this study has already been tested either on parathyroid cells and/or on HEK-CaSR cells (a model broadly used in the field).

Our study is a good example for testing the involvement of the CaSR in molecular processes. It is the first study, to our knowledge, that uses enantiomer pairs and combinations of positive and negative modulators to prove the involvement of a target in the signaling process in intestinal cells. Many natural (endogenous) ligands of the CaSR signal through numerous alternative pathways. Most of the pharmacological modulators have structures very similar to drugs acting on other molecules (e.g., Ca^2+^-channel or β-blockers). Therefore, it is difficult to exclude the possibility that the effects seen are mediated through other pathways, even more so when the concentrations used are higher than those needed to modulate CaSR activity. The *S* enantiomers could prove very useful in such cases, as they could be used to test if the effect seen is indeed caused by the binding of the drug to the CaSR.

The role of the CaSR in the gastrointestinal tract is complex. The clinical use of calcimimetics, both oral and intravenously applied, is associated with substantial gastrointestinal side effects, such as vomiting and nausea [11], and the U.S. Food and Drug Administration (FDA) has updated the label of cinacalcet to include the risk for upper gastrointestinal bleeding (FDA ID: 4097661). The actual, direct role of the gastrointestinal CaSR in (patho-)physiology and in these adverse effects is, however, still not fully understood. Several studies have shown that the CaSR is directly involved in intestinal fluid secretion [23,24]. However, the involvement of the CaSR in intestinal inflammation is still unclear [6]. The most frequent approaches to verify the role of CaSR in physiology and pathophysiology are either genetic, by comparing the phenotype of the mice lacking the CaSR to their wild type controls [25,26], or pharmacological approaches, using specific CaSR activators (e.g., *R-*568) or inhibitors (e.g., NPS 2143). One of the limitations of the genetic approach is that the organism might develop mechanisms to compensate for the loss of the CaSR. The limitations of the pharmacological approaches are that often the doses of the pharmacological compounds are too high [27], raising the question as to how this affects the specificity of the effect. The potent and selective pharmacological modulators of the CaSR (NPS *R-*568 and NPS *R-*2143) have been proven useful in uncovering new functions of the CaSR at different sites throughout the body [28]. However, it is important to use these compounds in a concentration that is selective to the CaSR, as at high concentrations, they will affect cellular responses independent of the CaSR [22]. In our study, we combined a genetic approach (but instead of knocking out, we introduced the CaSR in the cells) with the pharmacological approach, by using compounds at concentrations that are still specific for the CaSR and taking advantage of the stereospecificity of the used calcimimetic and calcilytic.

Our study demonstrates that in the HT29^CaSR-GFP^ cells, the major activators of the CaSR, Ca^2+^ and *R-*568, induced and did not inhibit the expression of the inflammatory cytokine IL-8. Our results unequivocally confirm the CaSR as a mediator of the enhanced inflammatory gene expression in these cells and are quadruple controlled: (1) a positive CaSR modulator induced inflammatory gene expression, (2) a negative CaSR modulator prevented this upregulation, (3) the unspecific enantiomers of these compounds did not exhibit these effects, and (4) none of these effects were observed in cells not expressing the CaSR.

These data also suggest that in native cells, where no endogenous CaSR is present, the drugs will not affect the expression of these inflammatory markers. This would be in line with our observation in an in vivo study, where neither the calcimimetic cinacalcet nor NPS *R-*2143 affected the expression of the inflammatory marker IL-6 in the colon of mice treated per gavage for 2 weeks with these modulators (unpublished results/manuscript in preparation). We now have a better understanding of the role of the CaSR in mediating inflammatory responses and with NPS *S*-2143, a valuable tool for controlling pharmacological CaSR modulation experiments

## 4. Materials and Methods

### 4.1. Preparation of NPS S-2143—Synthetic Chemistry

All solvents and reagents were used as obtained from commercial sources, unless otherwise indicated. All solvents used for chromatography were HPLC grade (Fisher Scientific, Loughborough, UK). All reactions were performed under a nitrogen atmosphere. ^1^H and ^13^C-NMR spectra were recorded with a Bruker Avance III HD spectrometer (Bruker, Coventry, UK) operating at 500 MHz for ^1^H and 125 MHz for ^13^C, with Me_4_Si as the internal standard. Deuterated chloroform was used as the solvent for NMR experiments. ^1^H chemical shifts values (δ) are referenced to the residual non-deuterated components of the NMR solvents (δ = 7.26 ppm for CHCl_3_, etc.). The ^13^C chemical shifts (δ) are referenced to CDCl_3_ (central peak, δ = 77.0 ppm). TLC was performed on silica gel 60 F254 plastic sheets. Normal-phase automated flash column chromatography was performed using a Biotage Isolera system (Biotage, Hengoed, UK). UPLC–MS analysis was conducted on a Waters UPLC system (Waters, Wilmslow, UK) with both Diode Array detection and Electrospray (+′ve and −′ve ion) MS detection. The stationary phase was a Waters Acquity UPLC BEH C18 1.7 um 2.1 × 50 mm column (Waters). The mobile phase was LC–MS grade H_2_O containing 0.1% formic acid (A) and LC–MS grade MeCN containing 0.1% formic acid (B). Column temperature: 40 °C. Sample diluent: MeCN or H_2_O. Sample concentration: 1 µg/mL. Injection volume: 2 µL. A linear gradient method was used for all analyses performed: 90% A (0.1 min), 90–0% A (1.5 min), 0% A (1.4 min), 90% A (0.1 min); flow rate: 0.5 mL/min. All compounds synthesised were >98% pure.

#### 4.1.1. Synthesis of (*S*)-2-chloro-6-(oxiran-2-ylmethoxy)benzonitrile **3**

Potassium carbonate (17.6 mmol, 3 eq.) was added to a stirring solution of 2-chloro-6-hydroxybenzonitrile **1** (5.9 mmol, 1 eq.) in dry acetone (60 mL), and the mixture was heated to reflux for 30 min. After cooling to room temperature, commercially available (*S*)-nosyl epoxide **2** (5.9 mmol, 1 eq.) was added to the reaction, and stirring was continued while heating to reflux overnight. After cooling to room temperature, the reaction mixture was filtered, and the filtrate was dried under vacuum. The crude residue was purified by flash column chromatography on silica gel (*n*-hexane/ethyl acetate 100:0 to *n*-hexane/ethyl acetate 0:100 *v*/*v*) to afford the pure title compound as a white solid in 98% yield. ^1^H-NMR (CDCl_3_), δ: 7.46 (dd, J_1_ = 8.9 Hz, J_2_ = 8.1 Hz, 1H), 7.12 (dd, J_1_ = 8.1 Hz, J_2_ = 0.8 Hz, 1H), 6.96 (dd, J_1_ = 8.9 Hz, J_2_ = 0.8 Hz, 1H), 4.42 (dd, J_1_ = 11.4 Hz, J_2_ = 2.8 Hz, 1H), 4.14 (dd, J_1_ = 11.4 Hz, J_2_ = 5.2 Hz, 1H), 3.43–3.40 (m, 1H), 2.96 (dd, J_1_ = 4.7 Hz, J_2_ = 4.1 Hz, 1H), 2.87 (dd, J_1_ = 4.7 Hz, J_2_ = 2.6 Hz, 1H). ^13^C-NMR (CDCl_3_), δ: 161.4, 138.1, 134.3, 122.3, 113.4, 110.7, 103.6, 69.8, 49.7, 44.5.

#### 4.1.2. Synthesis of (*S*)-2-chloro-6-(3-((2,2-dimethyl-3-(naphthalen-2-yl)propyl)amino)-2-hydroxypropoxy) benzonitrile (**NPS *S*-2143**)

A solution of (*S*)-epoxide **3** (3.3 mmol, 1 eq.) and [1,1-dimethyl-2-(2-naphthalenyl)ethyl]amine (3.3 mmol, 1 eq.) in dry EtOH (17 mL) was heated to 80 °C in a sealed tube for 72 h. The mixture was then dried under vacuum, and the crude residue was purified by flash column chromatography on silica gel (*n*-hexane/ethyl acetate 100:0 to *n*-hexane/ethyl acetate 0:100 *v*/*v*) to afford the pure title compound as a colourless oil in 73% yield. ^1^H-NMR (CDCl_3_), δ: 7.83–7.76 (m, 3H), 7.64 (s, 1H), 7.48–7.42 (m, 3H), 7.35 (dd, J_1_ = 8.3 Hz, J_2_ = 1.7 Hz, 1H), 7.10 (dd, J_1_ = 8.1 Hz, J_2_ = 0.8 Hz, 1H), 6.91 (dd, J_1_ = 7.8 Hz, J_2_ = 0.8 Hz, 1H), 4.18–4.12 (m, 2H), 4.03–3.98 (m, 1H), 3.06 (dd, J_1_ = 12.1 Hz, J_2_ = 4.6 Hz, 1H), 2.94–2.90 (m, 3H), 1.17 (s, 3H), 1.16 (s, 3H). ^13^C-NMR (CDCl_3_), δ: 137.7, 134.4, 133.2, 132.5, 131.8, 129.7, 128.7, 128.1, 127.7, 127.6, 126.3, 126.1, 122.4, 113.6, 110.9, 103.6, 71.2, 65.5, 61.5, 44.9, 44.4, 23.2, 23.0. UPLC–MS: R_t_ 1.65 min, MS [ESI, *m*/*z*]: 409.3, 411.3 [M + H].

#### 4.1.3. Synthesis of (*S*)-2-chloro-6-(3-((2,2-dimethyl-3-(naphthalen-2-yl)propyl)amino)-2-hydroxypropoxy) benzonitrile hydrochloride (**NPS *S*-2143.HCl**)

(*S*)-2-Chloro-6-(3-((2,2-dimethyl-3-(naphthalen-2-yl)propyl)amino)-2-hydroxypropoxy) benzonitrile (2.4 mmol, 1 eq.) was dissolved in MeOH (15 mL) and added dropwise of a concentrated HCl solution (3 mL), while stirring. Stirring was continued at room temperature for a further 1 h, and the residue was then dried under vacuum to afford the pure title compound as a white solid in quantitative yield. ^1^H-NMR (CDCl_3_), δ: 9.89 (bs, 1H), 8.37 (bs, 1H), 7.82–7.78 (m, 3H), 7.74 (s, 1H), 7.49–7.45 (m, 2H), 7.39–7.35 (m, 2H), 7.02 (d, J = 7.9 Hz, 1H), 6.91 (d, J = 8.5 Hz, 1H), 5.67 (d, J = 5.1 Hz, 1H), 4.84 (bs, 1H), 4.32–4.26 (m, 2H), 3.57–3.51 (m, 1H), 3.49–3.37 (m, 3H), 1.54 (s, 3H), 1.51 (s, 3H). ^13^C-NMR (CDCl_3_), δ: 161.8, 137.9, 135.8, 134.2, 133.3, 132.1, 129.2, 128.8, 127.5, 127.4, 125.9, 125.4, 122.0, 113.5, 110.5, 103.6, 71.9, 67.9, 53.7, 47.8, 43.9, 27.2, 27.1. UPLC–MS: R_t_ 1.70 min, MS [ESI, *m*/*z*]: 409.3, 411.3 [M + H].

### 4.2. Crystal Structure Determination

Single-crystal XRD data were collected at room temperature on an Agilent SuperNova Dual Atlas diffractometer (Agilent Technologies XRD Products, Yarnton, UK) with a mirror monochromator using Cu (λ = 1.5418 Å) radiation. The crystal structure was solved using SHELXS [29] and refined using SHELXL2018 [30]. Non-hydrogen atoms were refined with anisotropic displacement parameters, and hydrogen atoms were inserted in idealized positions. A riding model was used with Uiso(H) set at 1.2 or 1.5 times the Ueq(C,N,O) values of the atoms to which the H atoms are bonded. The asymmetric unit contains two cations and two chloride anions. The naphthalene moieties of both independent cations were modelled as disordered, and the components refined to roughly equal occupancy. The crystallographic and refinement parameters are as follows: C_24_H_26_ClN_2_O_2_^+^Cl^-^: FW = 445.37, T = 293(2) K, orthorhombic, P2_1_2_1_2_1_, a = 6.9104(2) Å, b = 16.3499(5) Å, c = 40.5957(9) Å, V = 4586.7(2) Å^3^, Z = 8, ρ_cal_ = 1.290 Mg/m^3^, μ = 2.722 mm⁻^1^, F(000) = 1872, crystal size = 0.575 × 0.114 × 0.039 mm, reflections collected = 43002, independent reflections = 9164, R(int) = 0.0629, goodness-of-fit on F^2^ = 1.016, Flack parameter = −0.009(8), largest diff. peak and hole = 0.258 and −0.256 e.Å⁻^3^, R1 = 0.0522 and wR2 = 0.1311 for I > 2σ(I), R1 = 0.0704 and wR2 = 0.1410 for all data.

### 4.3. Other Compounds and Reagents

NPS *R-*568 and NPS *R-*2143 were obtained commercially (Tocris Bioscience/Bio-Techne Ltd., Abingdon, UK), NPS *S-*568 was a kind gift from Amgen, UK. All basic compounds were obtained from Merck (Darmstadt, DE), unless otherwise stated.

### 4.4. Calcium Imaging Experiments

HEK293 cells stably transfected with the human CaSR (HEK-CaSR, a kind gift of Dr. Donald Ward [31]) were cultured on poly-D-lysine coated 13 mm coverslips, and calcium imaging experiments were performed as described previously [21]. In brief, the medium of the cells was removed, and cells were loaded with 3 µmol/L Fura 2-AM (Thermo Fisher Scientific, Waltham, MA, USA) in extracellular buffer containing 1.0 mmol/L Ca^2+^ for 45 min at 37 °C. Cells were then washed with ECS at room temperature and pre-incubated with buffer containing 0.2 mmol/L Ca^2+^ and 100 nmol/L NPS R/S-2143 or 0.1% DMSO for 15 min. Cells were imaged on an inverted Olympus IX71 fluorescence microscope (Olympus, Southend-on-Sea, UK). A rapid perfusion system was then used to alter extracellular Ca^2+^ from 0.2 (2 min, baseline) to 5 (3 min, stimulation) to 0.2 mmol/L Ca^2+^ (2 min), with each buffer containing either 100 nmol/L NPS *R/S*-2143 or 0.1% DMSO. Fluorescence of individual cells (~30–70 per experiment) at 340 and 380 nm was acquired every 2 s. For analysis, the background fluorescence was subtracted for both wavelengths, and the average F340/F380 ratio of the baseline of each cell was then subtracted from the maximum response of each cell during the stimulation phase. The average of these differences in one experiment counted as one biological repetition.

### 4.5. Colon Cancer Cells

We used lentiviral stably transduced colon cancer cell lines HT29^CaSR-GFP^ and HT29^GFP^ [9]. The parent HT-29 cell line was obtained commercially (HTB-38™) from the American Type Culture Collection (ATCC, Manassas, VA, USA). Transduced cells were cultured in Dulbecco’s Modified Eagle’s Medium containing 10% fetal calf serum, 100 U/mL Pen-Strep, 2 mmol/L L-glutamine and 10 mmol/L Hepes. Of note, the medium by itself contains 1.8 mmol/L Ca^2+^, the complete (FCS supplemented) medium contains ~ 2 mmol/L Ca^2+^. To select for transduced cells, 0.5 µL/mL puromycin (all Thermo Fisher Scientific) was added to the cell medium. For the treatments, cells were seeded into 6-well plates and grown to 70–80% confluence. 24 h before the treatments, the medium was changed without adding puromycin to let cells recover. All cells were regularly tested negatively for mycoplasma.

### 4.6. Compound Treatments

70–80% confluent cells in 6-well plates were treated for 4 h with 1 µmol/L of the CaSR modulators alone or in combination, or 5 mmol/L Ca^2+^ (through addition of an 0.25 mol/L aqueous solution of CaCl_2_). All compounds were prepared as 2 mmol/L stock solutions in DMSO and were added either by themselves and supplemented with DMSO, or in combination so that DMSO concentration in the medium was 0.1% for all conditions. NPS *R/S*-2143 was always added 15 min prior to the addition of NPS *R/S*-568 (pre-incubation period). After incubation, the cells were washed with ice-cold phosphate buffered saline, and finally lysed in Trizol (Thermo Fisher Scientific) by vigorous pipetting for RNA isolation.

### 4.7. Reverse Transcription Real Time PCR (RT-qPCR)

RNA was isolated from Trizol treated cells according to the manufacturer’s protocol. RNA concentrations and purity were measured spectrophotometrically, and integrity was confirmed by gel electrophoresis. Reverse transcription of 1000 ng RNA per sample was performed using the High-Capacity cDNA Reverse Transcription kit (Thermo Fisher Scientific) according to the manufacturer’s instructions. RT-qPCR was performed on QuantStudio 12K Flex and QuantStudio 5 real time PCR systems (Thermo Fisher Scientific) with the following program: 2 min 50 °C, 10 min 95 °C, 40 × 15 s 95 °C to 1 min 60 °C, followed by melting curve analysis 55–95 °C), using Power SYBR Green PCR Master Mix (Thermo Fisher Scientific). Relative quantification of the samples was performed using the ΔΔCt method, using the average of two housekeeping genes (ribosomal protein lateral stalk subunit P0 [*RPLP0*] & β2-macroglocbulin [*B2m*]), human total RNA calibrator (Takara, Kusatsu, JP) as reference, and the following primer sequences: *RPLP0*: TGGTCATCCAGCAGGTGTTCGA (fwd), GCAGCAGCTGGCACCTTATTG (rev); *B2m*: GATGAGTATGCCTGCCGTGTG (fwd), CAATCCAAATGCGGCATCT (rev); *CaSR*: GCCAAGAAGGGAGAAAGAC (fwd), CACACTCAAAGCAGCAGG (rev); *IL-8*: CTTGGCAGCCTTCCTGATTT (fwd), TTCTTTAGCACTCCTTGGCAAAA (rev) [9].

### 4.8. Statistical Analysis

Statistical analysis and visualizations were performed used GraphPad Prism 9.2 (GraphPad Software, San Diego, CA, USA). The applied statistical tests are indicated in the captions of each figure.

## Data Availability

Supplementary crystallographic data are available at CCDC 2097408.

## Supplementary Materials

The following are available online at https://www.mdpi.com/article/10.3390/ijms221810124/s1.
