# Supplementary material for: Stereo-Specific Modulation of the Extracellular Calcium-Sensing Receptor in Colon Cancer Cells
Source: Int J Mol Sci. 2021 Sep 19;22(18):10124. doi: 10.3390/ijms221810124 (PMC8464956; doi:10.3390/ijms221810124)
Supplement: Supplementary file 1 [file ijms-22-10124-s001.zip › ijms-1356398-supplementary.pdf]

Supplementary Data

Stereo-specific modulation of the extracellular calcium-sensing receptor in colon cancer cells

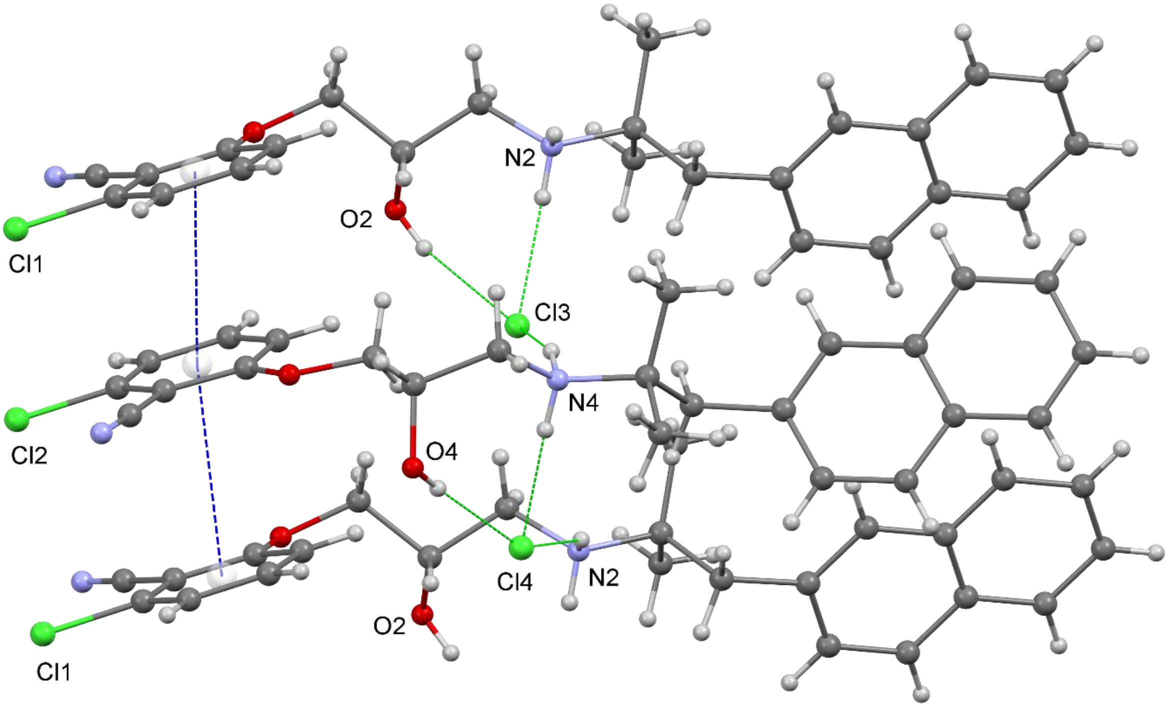

Figure S1: Intermolecular π-π (blue dotted lines) and hydrogen bonding contacts (green dotted lines) in the crystal structure of NPS S-2143.HCl.

Table S1: Selected torsion angles [°] in the crystal structure of NPS S-2143.HCl.

**(a)**		**(b)**	
C9-C8-O1-C1	79.(25)	C33-C32-O3-C25	-168.7(3)
O1-C8-C9-O2	61.3(5)	O3-C32-C33-O4	67.2(4)
O1-C8-C9-C10	-178.1(4)	O3-C32-C33-C34	-165.5(3)
C8-C9-C10-N2	158.5(4)	C32-C33-C34-N4	-65.4(5)
C9-C10-N2-C11	152.3(4)	C33-C34-N4-C35	-156.4(4)
C14-C11-N2-C10	174.0(4)	C38-C35-N4-C34	-176.3(4)
N2-C11-C14-C16	169.5(6)	N4-C35-C38-C40	-178.0(7)

Table S2: Hydrogen bonding contacts (D = N or O) in the crystal structure of NPS S-2143.HCl.

	D…Cl distance/Å	D-H…Cl angle/°
N2-H2A…Cl3	3.092(4)	166.1
N2-H2B…Cl4	3.199(4)	149.1
N4-H4A…Cl3	3.221(4)	157.2
N4-H4B…Cl4	3.162(3)	164.2
O2-H2…Cl3	3.256(3)	153.2
O4-H4C…Cl4	3.205(4)	175.1
